# Changes in long term survival after diagnosis with common hematologic malignancies in the early 21st century

**DOI:** 10.1038/s41408-020-0323-4

**Published:** 2020-05-13

**Authors:** Dianne Pulte, Lina Jansen, Hermann Brenner

**Affiliations:** 1 Division of Clinical Epidemiology and Aging Research, German Cancer Research Center (DKFZ), Heidelberg, Germany; 2 Division of Preventive Oncology, German Cancer Research Center (DKFZ) and National Center for Tumor Diseases (NCT), Heidelberg, Germany; 3 German Cancer Consortium (DKTK), German Cancer Research Center (DKFZ), Heidelberg, Germany

**Keywords:** Epidemiology, Epidemiology

## Abstract

Five-year survival has increased for many hematologic malignancies in the 21st century. However, whether this has translated into greater long-term survival is unknown. Here, we examine 10- and 20-year survival for patients with multiple myeloma (MM), acute lymphoblastic leukemia (ALL), acute myeloblastic leukemia (AML), chronic lymphoid leukemia (CLL), chronic myeloid leukemia (CML), non-Hodgkin lymphoma (NHL), and Hodgkin lymphoma (HL). Data were extracted from the Surveillance, Epidemiology, and End Results-9 database. Patients age 15+ with the above malignancies were included. The newly developed boomerang method was used to examine 10- and 20-year relative survival (RS) for patients in 2002–2006 and 2012–16. Ten and 20-year RS increased for each malignancy examined, with increases ranging from +4.4% units for 20-year RS for AML to +23.1% units for 10-year RS for CML. Ten year RS was >50% in 2012–16 for patients with CLL, CML, HL, NHL, and DLBCL, at 77.1%, 62.1%, 63.9%, 64.5%, and 63.0%, respectively. Survival dropped between 10 and 20 years after diagnosis for most malignancies. Long-term survival is increasing for common hematologic malignancies, but late mortality is an ongoing issue. Further study of long-term outcomes in curable malignancies to determine the reason for these later decreases in survival is indicated.

## Background

Recent progress in the treatment of many hematologic malignancies has resulted in improved population level 10-year survival^1^, particularly chronic myeloid leukemia (CML)^2,3^, chronic lymphocytic leukemia (CLL)^4^, multiple myeloma (MM)^5,6^, and some subtypes of non-Hodgkin lymphoma (NHL)^7^. However, how these changes have effected longer term survival on the population level is less well documented.

Documentation of changes in longer term survival is hampered by the fact that improved survival for many malignancies has occurred only due to changes in therapy that may have occurred only in the past decade and analysis of longer term survival will necessarily include data from patients treated prior to the availability of these therapies. For example, tyrosine kinase inhibitors (TKI) drastically changed the natural history of CML and the first TKI was approved in the United States (US) only in 2001^8^. Thus, any estimate of, for example, 20 year survival will necessarily be an underestimate, even using period analysis, a method that has been shown to provide more up-to-date survival estimates than traditional cohort-based survival analysis methods^9^. Recently, a new method of survival analysis, the boomerang method^10^, was proposed to further enhance up-to-dateness of long-term survival estimates. This method was shown empirically to provide long-term survival estimates closer to those later observed when survival is changing rapidly, i.e. when survival improves drastically due to new therapeutic options.

Here, we examine 10- and 20-year survival for patients with common hematologic malignancies in the United States (US) using the boomerang method.

## Methods

Data were extracted from the Surveillance, Epidemiology, and End Results (SEER)9 database. The SEER9 database includes data from nine regional cancer registries throughout the US. Registries are chosen for their high quality and epidemiologically significant populations^11^. The SEER9 registries provide data on cancer survival starting in 1973 and thus are ideal sources of data for examination of long term population level survival. The population within the SEER registry is similar to the general US population in most respects, although there is deliberate oversampling of some minority ethnic groups and a higher proportion of foreign-born persons than in the general US population^11^. Adults with a diagnosis of myeloma, acute lymphoblastic leukemia (ALL), acute myeloblastic leukemia (AML), CLL, CML, Hodgkin lymphoma (HL) and NHL selected by ICD-10 codes C90.0 (MM), C91.0 (ALL), C92.0, C92.3, C92.4, C92.5, C92.6, C92.8, and C92.9 (AML), C91.1 (CLL), C92.1 (CML), C81 (HL), and C82-86 (NHL), were included in the analysis. Because NHL is a very heterogeneous condition with multiple subtypes with varying survival expectations, an analysis on the most common type of NHL, diffuse large B-cell lymphoma (DLBCL) was performed as well, with cases selected using the ICD-10 code C83.3. Cases diagnosed by death certificate only (DCO) were excluded.

Ten and twenty-year survival for patients diagnosed with the above malignancies in 2002–2006 and 2012–16 were estimated using the boomerang method. The boomerang method of estimating survival has been described in detail elsewhere^10^. Briefly, with the boomerang approach, the survival experience in the initial 5 years for a given time of diagnosis is obtained by a complete analysis of survival for patients diagnosed in the most recent calendar years, whereas survival for later years (i.e., 6–10 or 6–20 years) after diagnosis is contributed by the survival experience diagnosed in earlier years in a “period-like” approach, minimizing the contribution of the survival experience of patients diagnosed in earlier calendar periods (see Supplemental Fig. 1). This method has been demonstrated to produce survival estimates closer to those later observed for cancers where survival is changing rapidly, i.e. those where new treatment options have changed the survival expectations.

According to common practice in population-based cancer survival studies, relative, rather than absolute, survival was assessed. Relative survival (RS) estimates are obtained by taking the ratio of absolute survival rates compared to the expected survival rates for a similar group of people in the general population. Expected survival was derived using age-, sex-, and race-specific life tables from the United States^12^, using the Ederer II method^13^. Age standardized survival was calculated using International Cancer Survival Standard (ICSS) weights^14^ for 5 age groups. In addition, when possible, age-specific survival was calculated for two age groups: 15–64 and 65+ years of age.

Survival estimates for which the standard error was greater than 5 percent units due to sparseness of data are not reported. Standard errors of greater than 5% units limited the time period for which results could be reported for patients with CLL, CML, HL, and DLBCL. In addition, analysis of older patients was not conducted for ALL and CML due to the very small case numbers leading to high standard errors. Past analyses of survival for patients with HL have shown extreme variation in survival between young people with HL and older patients^15^. Possibly because of this variation, standard errors for HL overall were too high to calculate survival estimates after 10 years for all patients and for any time interval for older adults, although standard errors were actually lower for younger patients, possibly due to less heterogeneity, allowing for calculation of up to 20-year survival in this patient population.

All analyses were run on SAS Software (version 9.4, SAS, Carey, NC, USA) using macros for boomerang analyses as previously described^10^.

## Results

Case numbers after the exclusion of DCO cases ranged from 952 cases of ALL in 2002–06 to 30,333 cases of NHL in 2012–16 (Table 1). The overall DCO rate was 0.7–1.2% for leukemias, 1.6% for MM, and 0.2–0.8% for lymphomas, with no individual histologies with a DCO rate over 2%. Case numbers increased in the later period as compared to the earlier for all malignancies. MM, CLL, and NHL were the most common malignancies in each time period, CML and ALL the least common. More men than women were diagnosed with hematologic malignancies in the relevant time periods for all malignancies examined. There was little change in gender distribution over time. As expected, median age at diagnosis was lowest for ALL and HL, highest for myeloma and CLL. The median age at diagnosis changed by one year or less between the two time periods for all conditions except for patients with CLL, for whom the median age decreased by 2 years, and for patients with ALL, for whom the median age at diagnosis increased by 4 years.

**Table 1:** Case numbers by histology and calendar period after removal of cases diagnosed by death certificate only (DCO) and proportion of DCO cases for 2002–2016.

Histology	Category	2002–2006	2012–2016	% DCO
Myeloma	All	7508	10,782	1.6%
	Gender			
	M	4049 (54%)	6076 (56%)	
	F	3459 (46%)	4706 (44%)	
	Median age	70	69	
ALL	All	952	1384	0.7%
	Gender			
	M	555 (58%)	805 (58%)	
	F	397 (42%)	579 (42%)	
	Median Age	45	49	
AML	All	4611	6901	1.1%
	Gender			
	M	2441 (53%)	3781 (55%)	
	F	2170 (47%)	3120 (45%)	
	Median Age	68	69	
CLL	All	7278	10,137	1.0%
	Gender			
	M	4334 (60%)	6212 (61%)	
	F	2944 (40%)	3925 (39%)	
	Median Age	71	69	
CML	All	1621	2220	1.2%
	Gender			
	M	931 (57%)	1254 (56%)	
	F	690 (43%)	966 (44%)	
	Median Age	61	60	
HL	All	3877	3918	0.4%
	Gender			
	M	2073 (53%)	2213 (56%)	
	F	1804 (47%)	1705 (44%)	
	Median Age	39	40	
NHL	All	27,555	30,333	0.8%
	Gender			
	M	14,785 (54%)	16,762 (55%)	
	F	12,770 (46%)	13,571 (45%)	
	Median Age	67	67	
DLBCL	All	11,927	15,079	0.2%
	Gender			
	M	6206 (52%)	8122 (54%)	
	F	5721 (48%)	6957 (46%)	
	Median Age	68	68	

*ALL* acute lymphoblastic leukemia, *AML* acute myeloblastic leukemia, *CLL* chronic lymphoid leukemia, *CM* chronic myeloid leukemia, *HL* Hodgkin lymphoma, *NHL* non-Hodgkin lymphoma, *DLBCL* diffuse large B-cell lymphoma.

**Fig. 1:**
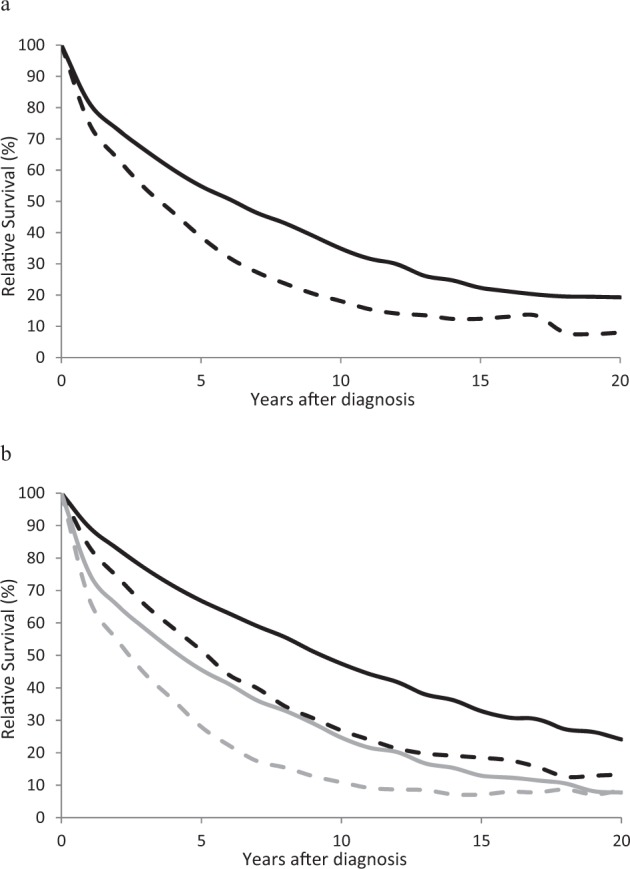
Age-adjusted and age-specific long term relative survival for patients with multiple myeloma. **a** Age-adjusted 0–20 relative survival for 2002–2006 (dashed line) and 2012–2016 (solid line.) **b** Age-specific 0–20-year relative survival for patients age 15–64 2002–2006 (black dashed line), 15–64 2012–2016 (black solid line), 65+ 2002–2006 (gray dashed line) and 2012–2016 (gray solid line).

Ten and twenty-year age-standardized RS for patients with MM increased from 18.1% and 8.0%, respectively, in 2002–2006 to 34.9% and 19.3%, respectively, in 2012–16 (Fig. 1a, Table 2). Survival was greater at both time points for patients age 15–64, with an increase of +20.5% units and +10.8% units at 10 and 20 years, respectively (Fig. 1b, Table 2). Both survival and changes in survival between the two-time points were lower for older patients, but an increase in RS was observed at the 10-year time point.

**Table 2:** Ten and 20 year relative survival for patients with hematologic malignancies by malignancy, age, and period.

Histology	Population	2002–06		2012–16	
		10-year RS (SE)	20-year RS (SE)	10-year RS (SE)	20-year RS (SE)
Myeloma	All	18.1 (1.3)	8.0 (1.8)	34.9 (1.4)	19.3 (4.4)
	15–64	26.9 (2.3)	13.3 (2.9)	47.4 (2.1)	24.1 (3.1)
	65+	10.9 (1.4)	8.4 (3.8)	24.7 (1.9)	7.8 (3.2)
ALL	All	13.0 (2.2)	5.6 (1.3)	29.0 (3.9)	16.5 (2.6)
	15–64	36.8 (3.2)	34.9 (3.8)	47.0 (3.2)	43.8 (3.9)
AML	All	16.1 (1.2)	10.1 (1.5)	19.0 (1.0)	14.5 (1.7)
	15–64	31.1 (1.9)	29.8 (2.4)	39.6 (1.6)	33.9 (2.5)
	65+	6.2 (1.3)	0	6.1 (1.1)	4.9 (2.5)
CLL	All	67.8 (2.3)	37.3 (3.1)	77.1 (1.8)	55.9 (4.2)
	15–64	73.8 (2.7)	51.2 (4.4)	85.8 (1.8)	73.8 (3.8)
	65+	63.8 (3.2)	NA	70.8 (2.6)	NA
CML	All	39.0 (3.7)	NA	62.1 (3.6)	NA
	15–64	69.2 (3.9)	NA	84.7 (2.4)	NA
HL	All	50.6 (3.5)	NA	63.9 (4.0)	NA
	15–64	84.9 (1.3)	77.8 (1.9)	88.7 (1.2)	82.6 (1.8)
NHL	All	56.5 (1.0)	41.5 (3.5)	64.5 (0.9)	52.2 (2.7)
	15–64	68.4 (0.9)	54.6 (1.6)	75.6 (0.8)	69.4 (1.3)
	65+	48.6 (1.5)	35.0 (3.9)	56.8 (1.4)	42.0 (3.4)
DLBCL	All	56.9 (1.8)	NA	63.0 (1.3)	NA
	15–64	68.6 (1.5)	57.2 (2.8)	72.1 (1.2)	64.8 (2.0)
	65+	48.6 (2.7)	NA	56.3 (2.0)	NA

**Fig. 2:**
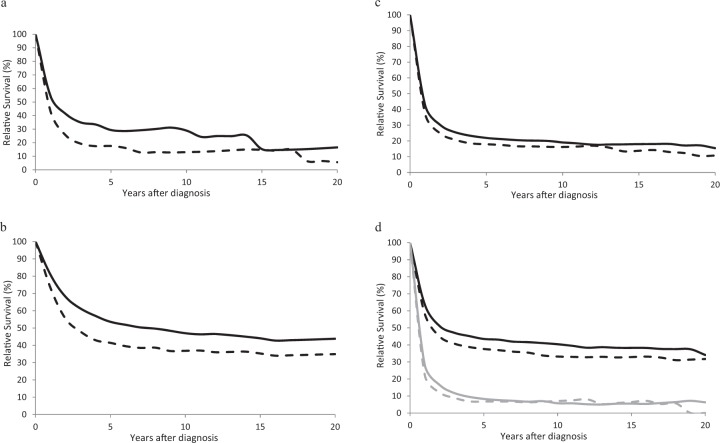
Age-adjusted and age-specific long term relative survival for patients with acute leukemia. **a** Age-adjusted relative survival for patients with ALL in 2002–2006 (dashed line) and 2012–2016 (solid line). **b** Relative survival for patients age 15–64 with ALL in 2002–2006 (dashed line) and 2012–2016 (solid line). **c** Age-adjusted 0–20-year survival for patients with AML in 2002–2006 (dashed line) and 2012–2016 (solid line). **d** Age-specific 0–20-year relative survival for patients with AML age 15–64 2002–2006 (black dashed line) and 2012–2016 (black solid line) and for patients age 65+ in 2002–2006 (gray dashed line) and 2012–2016 (gray solid line).

Relative survival was low for patients with ALL, with 10- and 20-year estimates of 13.0% and 5.6%, respectively, for 2002–2006 and 29.0% and 16.5% for 2012–2016 (Fig. 2a, Table 2). However, RS was better and changes in RS between the two time points greater for patients age 15–64 (Fig. 2b, Table 2). Survival for younger patients reached a near plateau after 5 years, with only a small decrease in relative survival between 5 and 10 years and between 10 and 20 years in 2012–2016.

Ten year RS for patients with AML increased from 14.0% in 2002–2006 to 19.0% in 2012–16 (Fig. 2c, Table 2). A small but persistent decrease in survival was observed between 10 and 20 years for both time periods. Survival varied considerably by age, with 10-year survival estimates for patients age 15–64 of 31.1% in 2002–2006 and 39.6% in 2012–2016 as compared to 10-year estimates of 6.2% for 2002–2006 and 6.1% in 2012–2016 for age 65+ (Fig. 2d, Table 2). A decrease in RS was observed between 10 and 20 years for younger patients.

**Fig. 3:**
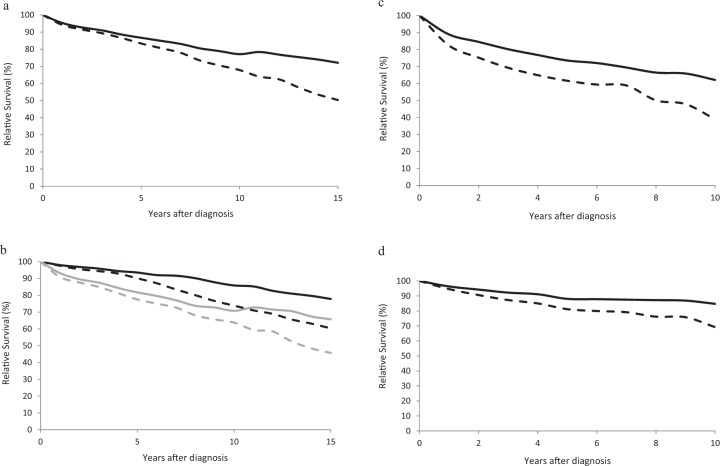
Age-adjusted and age-specific long term relative survival for patients with chronic leukemia. **a** Age-adjusted 0–20 relative survival for patients with CLL in 2002–2006 (dashed line) and 2012–2016 (solid line). **b** Age-specific 0–20-year relative survival for patients with CLL age 15–64 in 2002–2006 (black dashed line) and 2012–2016 (black solid line) and for patients age 65+ in 2002–2006 (gray dashed line) and 2012–2016 (gray solid line). **c** Age-adjusted 0–20-year survival for patients with CML in 2002–2006 (dashed line) and 2012–2016 (solid line). **d** Age-specific 0–10-year relative survival for patients with CML age 15–64 in 2002–2006 (black dashed line) and 2012–2016 (black solid line) and for patients age 65+ in 2002–2006 (gray dashed line) and 2012–2016 (gray solid line).

For patients with CLL, a major increase in RS was observed, with 10-year survival increasing by +9.3% units and 15-year survival increasing by18.6% units (Fig. 3a, Table 2). Survival estimates were higher for patients age 15–64 but increases in survival were observed for both younger and older patients. (Fig. 3b, Table 2), with 15-year relative survival going from 60.3% in 2002–2006 to 77.8% in 2012–2016 (+17.5% units) for patients age 15–64 and from 45.7% to 65.7% (+20% units) for patients age 65+.

Changes in survival were observed for CML, with 10-year estimates of RS going from 39.0% in 2002–2006 to 62.1% (+23.1% units) in 2012–2016 (Fig. 3c, Table 2). For patients age 15–64, 10 year relative survival in 2002–2006 was 69.2% and increased to 84.7% in 2012–2016 (Fig. 3d, Table 2).

**Fig. 4:**
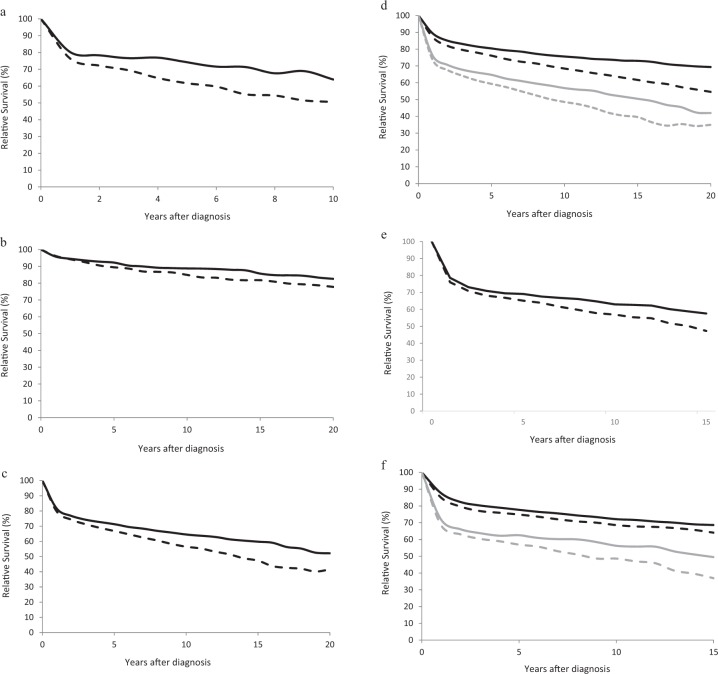
Age-adjusted and age-specific long term relative survival for patients with HL and NHL. **a** Relative survival for patients with HL in 2001–2005 (dashed line) and 2012–2016 (solid line). **b** Relative survival for patients age 15–64 with HL in 2002–2006 (dashed line) and 2012–2016 (solid line). **c** Age-adjusted 0–20 year survival for patients with NHL in 2002–2006 (dashed line) and 2012–2016 (solid line). **d** Age-specific 0–20-year relative survival for patients with NHL age 15–64 in 2002–2006 (black dashed line) and 2012–2016 (black solid line) and for patients age 65+ in 2002–2006 (gray dashed line) and 2012–2016 (gray solid line). **e** Age-adjusted 0–20-year survival for patients with DLBCL in 2002–2006 (dashed line) and 2012–2016 (solid line). **f** Age-specific 0–20-year relative survival for patients with DLBCL age 15–64 in 2002–2006 (black dashed line) and 2012–2016 (black solid line) and for patients age 65+ in 2002–2006 (gray dashed line) and 2012–2016 (gray solid line).

One to 10-year and a subgroup analysis of patients age 15–64 were calculated for HL. The overall 10-year analysis showed survival of 50.6% at 10 years in 2002–06 and 63.9% at 10 years in 2012–2016 (Fig. 4a, Table 2). Subgroup analysis of patients age 15–64 showed 10- and 20-year RS estimates of 84.9% and 77.8% for 2002–2006 and 88.7% and 82.6% for 2012–2016 (Fig. 4b, Table 2).

Survival for patients with NHL also demonstrated a large increase between the two times examined (Fig. 4c, Table 2). Survival estimates for the later time period were greater for both older and younger patients on age-specific analysis (Fig. 4d, Table 2).

Because there are multiple subtypes of NHL with varying prognosis, survival for diffuse large B-cell lymphoma (DLBCL), the most common subtype, was calculated as well (Fig. 4e, Table 2). An increase in survival for patients with DLBCL of +7.8% units and +10.3% units at 10 and 15 years, respectively, was observed. RS at 10 and 15 years in 2012–2016 were 63.0 and 57.6%. Survival increased for both younger and older patients between the two time periods, with a greater change for older patients (Fig. 4f, Table 2). For both patient groups, a rapid drop in survival the first several years after diagnosis was followed by a slow but persistent decrease in RS up to 15 years after diagnosis.

## Discussion

This first comprehensive application of the newly introduced boomerang method for up-to-date long-term survival estimates showed that overall 10- and 20-year survival increased substantially for each of the relatively common hematologic malignancies examined. Particularly impressive increases were observed for patients with CLL, CML, and NHL. However, 20-year relative survival remains under 50% for most of the malignancies examined, with the notable exception of HL in younger patients and plateaus in survival were observed only in a minority of cases and were not observed for NHL, in particular for DLBCL, which is generally considered curable with treatment.

A remarkable number of new treatment options have become available for the treatment of hematologic malignancies in the early 21st century, with particularly strong progress being made for MM^16^, CLL^17^, CML^18^, and NHL^19^, though the past decade has also seen the advent of new therapeutic options for ALL^20^, HL^21^, and some forms of AML^22^. Five year RS is now greater than 50% for all of these malignancies except for the acute leukemias^1–7,15,23,24^ and even for these conditions, 5-year survival greater than 50% for younger patients is not unexpected^25,26^. Five year RS expectations for younger patients with CML and HL as well as all patients with CLL are near to or greater than 90%^2–4,15,26^. Thus, examination of longer outcomes and the late effects of treatment are becoming more important as more patients survive the initial phase of their disease.

One notable finding is that for all conditions examined except for AML and arguably ALL in younger patients, there was a major decrease in survival between 10 and 20 years after diagnosis. This is seen even in NHL which is considered curable and thus little or no further excess mortality would be expected after the initial loss of life in the first few years after diagnosis and CML, which is generally thought to be controllable with relatively little loss of life expectancy if initial control of the disease can be established^27^. This decrease may be partly related to an underestimation of survival due to the advent of new therapeutic options that were not available during the earlier time periods necessarily included in the survival calculation even with the boomerang method. However, a recent publication found a risk of late relapse for patients with DLBCL, suggesting that some of the decrease in survival observed may be related to late relapse^28^. Taken together, these results suggest that a component of late relapse or increased risk of mortality from other causes, including late effects of treatment, may contribute to the decreased long term survival even in cancers that may be curable. Given the good short term prognosis of many hematologic malignancies, consideration of long term toxicities should be a part of treatment planning for patients starting in the first line.

Prior studies in HL have demonstrated that survivors of HL have an increased risk of cardiac disease and other malignancies^29^. In addition, an increased risk of secondary malignancy has been observed in patients with MM, especially after treatment with immunomodulatory agents^30^. Thus, it is likely that some of the decrease in survival between 10 and 20 years observed with most malignancies is related to increased risk of other causes of mortality. Increased monitoring and aggressive screening of survivors of hematologic malignancies for conditions where screening has been demonstrated to reduce mortality or morbidity and is appropriate for the specific patient may help decrease this excess mortality. In addition, while continued efforts to find curative treatment for these malignancies is important, an increased focus on potential long term sequelae of treatment, i.e. the potential for increased risk of cancer due to DNA damaging chemotherapy or of heart disease due to radiation to the chest, must be considered when evaluating potential treatment regimens.

One unexpected finding was that relative survival estimates did not always decrease consistently with time. For example, patients with ALL had an apparent increase in relative survival at about 9 and 14 years follow-up in the 2012–16 time period. This apparent increase in relative survival is likely due to small case numbers and statistical “noise”. It is possible, but less likely, that better than average medical monitoring or better health habits in survivors of cancer led to a decreased risk of death due to other causes at some time points.

Strengths of our study include the use of the large and long running SEER database, which allows for examination of long term survival in patients with hematologic malignancies, most of which are relatively rare. In addition, the use of the boomerang method allows for estimates of survival that are expected to be much closer to later observed survival experience of recently diagnosed patients compared to the use of older methods such as cohort analysis, in a condition where survival is changing rapidly.

In considering our results, some limitations should be kept in mind. First, the SEER database does not include information on chemotherapy use and whether treatment was provided with curative or palliative intent, so any correlation between changes in therapy and survival is necessarily indirect. Second, the SEER database does not include other information of potential prognostic significance such as white blood cell count at diagnosis for acute leukemia or prognostic score for NHL. Therefore, it is not possible to state definitively that there have been no changes in these factors at diagnosis which might influence outcomes. Third, even with the use of the boomerang method, it is likely that long term survival estimates are lower than the actual survival of patients diagnosed in the current era due to the necessity of including patients diagnosed before the advent of many modern therapeutic agents. Fourth, even in the 3 years between the most recent data in SEER and the present, a number of new therapeutic options have become available and thus the full effect of changes in therapy on the population level is not yet evident. Finally, in this paper, we did not consider subgroup analyses of potential importance (i.e., race, gender, and cause of death). In addition, the reasons for late mortality may be changing as treatment shifts from use of cytotoxic chemotherapy only to use of combination cytotoxic therapy, immune therapy, and small molecule enzyme inhibitors.

In summary, 10- and 20-year RS has substantially increased for common hematologic malignancies between 2002–2006 and 2012–2016. Long-term survival is still low and no plateau in survival was observed for most conditions, suggesting that there may be continued late mortality, either from late effects of the disease or therapy. Further research to determine the etiology of these issues and any changes over time in the causes of late mortality as well as to minimize therapeutic toxicity is needed to ensure optimal outcomes.

## Supplementary information

Reporting checklist

Supplemental Material
