# Supplementary material for: Changes in long term survival after diagnosis with common hematologic malignancies in the early 21st century
Source: Blood Cancer J. 2020 May 13;10(5):56. doi: 10.1038/s41408-020-0323-4 (PMC7221083; doi:10.1038/s41408-020-0323-4)
Supplement: Supplementary file 2 — Supplemental Material [file 41408_2020_323_MOESM2_ESM.docx]

Supplemental figure 1a: Graphic representation of boomerang analysis for 10-year survival for patients diagnosed in 2012-16. Numbers within cells indicate the years of follow-up after diagnosis. Only survival experience in the grey shadowed cells is included in the analysis.

| Years of diagnosis | Years of follow-up | | | | | | | | | | | | | | |
| --- | --- | --- | --- | --- | --- | --- | --- | --- | --- | --- | --- | --- | --- | --- | --- |
|  | 2002 | 2003 | 2004 | 2005 | 2006 | 2007 | 2008 | 2009 | 2010 | 2011 | 2012 | 2013 | 2014 | 2015 | 2016 |
| 2002 | 1 | 1-2 | 2-3 | 3-4 | 4-5 | 5-6 | 6-7 | 7-8 | 8-9 | 9-10 | 10 |  |  |  |  |
| 2003 |  | 1 | 1-2 | 2-3 | 3-4 | 4-5 | 5-6 | 6-7 | 7-8 | 8-9 | 9-10 | 10 |  |  |  |
| 2004 |  |  | 1 | 1-2 | 2-3 | 3-4 | 4-5 | 5-6 | 6-7 | 7-8 | 8-9 | 9-10 | 10 |  |  |
| 2005 |  |  |  | 1 | 1-2 | 2-3 | 3-4 | 4-5 | 5-6 | 6-7 | 7-8 | 8-9 | 9-10 | 10 |  |
| 2006 |  |  |  |  | 1 | 1-2 | 2-3 | 3-4 | 4-5 | 5-6 | 6-7 | 7-8 | 8-9 | 9-10 | 10 |
| 2007 |  |  |  |  |  | 1 | 1-2 | 2-3 | 3-4 | 4-5 | 5-6 | 6-7 | 7-8 | 8-9 | 9-10 |
| 2008 |  |  |  |  |  |  | 1 | 1-2 | 2-3 | 3-4 | 4-5 | 5-6 | 6-7 | 7-8 | 8-9 |
| 2008 |  |  |  |  |  |  |  | 1 | 1-2 | 2-3 | 3-4 | 4-5 | 5-6 | 6-7 | 7-8 |
| 2010 |  |  |  |  |  |  |  |  | 1 | 1-2 | 2-3 | 3-4 | 4-5 | 5-6 | 6-7 |
| 2011 |  |  |  |  |  |  |  |  |  | 1 | 1-2 | 2-3 | 3-4 | 4-5 | 5-6 |
| 2012 |  |  |  |  |  |  |  |  |  |  | 1 | 1-2 | 2-3 | 3-4 | 4-5 |
| 2013 |  |  |  |  |  |  |  |  |  |  |  | 1 | 1-2 | 2-3 | 3-4 |
| 2014 |  |  |  |  |  |  |  |  |  |  |  |  | 1 | 1-2 | 2-3 |
| 2015 |  |  |  |  |  |  |  |  |  |  |  |  |  | 1 | 1-2 |
| 2016 |  |  |  |  |  |  |  |  |  |  |  |  |  |  | 1 |

Supplemental figure 1b: Graphic representation of the boomerang method for 20-year survival for the years 2012-16. Numbers within cells indicate the years of follow-up after diagnosis. Only survival experience in the grey shadowed cells is included in the analysis.

| Years of diagnosis | Years of follow-up | | | | | | | | | | | | | | | | | | | | |
| --- | --- | --- | --- | --- | --- | --- | --- | --- | --- | --- | --- | --- | --- | --- | --- | --- | --- | --- | --- | --- | --- |
|  | 1996 | 1997 | 1998 | 1999 | 2000 | 2001 | 2002 | 2003 | 2004 | 2005 | 2006 | 2007 | 2008 | 2009 | 2010 | 2011 | 2012 | 2013 | 2014 | 2015 | 2016 |
| 1996 | 1 | 1-2 | 2-3 | 3-4 | 4-5 | 5-6 | 6-7 | 7-8 | 8-9 | 9-10 | 10-11 | 11-12 | 12-13 | 13-14 | 14-15 | 15-16 | 16-17 | 17-18 | 18-19 | 19-20 | 20 |
| 1997 |  | 1 | 1-2 | 2-3 | 3-4 | 4-5 | 5-6 | 6-7 | 7-8 | 8-9 | 9-10 | 10-11 | 11-12 | 12-13 | 13-14 | 14-15 | 15-16 | 16-17 | 17-18 | 18-19 | 19-20 |
| 1998 |  |  | 1 | 1-2 | 2-3 | 3-4 | 4-5 | 5-6 | 6-7 | 7-8 | 8-9 | 9-10 | 10-11 | 11-12 | 12-13 | 13-14 | 14-15 | 15-16 | 16-17 | 17-18 | 18-19 |
| 1999 |  |  |  | 1 | 1-2 | 2-3 | 3-4 | 4-5 | 5-6 | 6-7 | 7-8 | 8-9 | 9-10 | 10-11 | 11-12 | 12-13 | 13-14 | 14-15 | 15-16 | 16-17 | 17-18 |
| 2000 |  |  |  |  | 1 | 1-2 | 2-3 | 3-4 | 4-5 | 5-6 | 6-7 | 7-8 | 8-9 | 9-10 | 10-11 | 11-12 | 12-13 | 13-14 | 14-15 | 15-16 | 16-17 |
| 2001 |  |  |  |  |  | 1 | 1-2 | 2-3 | 3-4 | 4-5 | 5-6 | 6-7 | 7-8 | 8-9 | 9-10 | 10-11 | 11-12 | 12-13 | 13-14 | 14-15 | 15-16 |
| 2002 |  |  |  |  |  |  | 1 | 1-2 | 2-3 | 3-4 | 4-5 | 5-6 | 6-7 | 7-8 | 8-9 | 9-10 | 10-11 | 11-12 | 12-13 | 13-14 | 14-15 |
| 2003 |  |  |  |  |  |  |  | 1 | 1-2 | 2-3 | 3-4 | 4-5 | 5-6 | 6-7 | 7-8 | 8-9 | 9-10 | 10-11 | 11-12 | 12-13 | 13-14 |
| 2004 |  |  |  |  |  |  |  |  | 1 | 1-2 | 2-3 | 3-4 | 4-5 | 5-6 | 6-7 | 7-8 | 8-9 | 9-10 | 10-11 | 11-12 | 12-13 |
| 2005 |  |  |  |  |  |  |  |  |  | 1 | 1-2 | 2-3 | 3-4 | 4-5 | 5-6 | 6-7 | 7-8 | 8-9 | 9-10 | 10-11 | 11-12 |
| 2006 |  |  |  |  |  |  |  |  |  |  | 1 | 1-2 | 2-3 | 3-4 | 4-5 | 5-6 | 6-7 | 7-8 | 8-9 | 9-10 | 10-11 |
| 2007 |  |  |  |  |  |  |  |  |  |  |  | 1 | 1-2 | 2-3 | 3-4 | 4-5 | 5-6 | 6-7 | 7-8 | 8-9 | 9-10 |
| 2008 |  |  |  |  |  |  |  |  |  |  |  |  | 1 | 1-2 | 2-3 | 3-4 | 4-5 | 5-6 | 6-7 | 7-8 | 8-9 |
| 2009 |  |  |  |  |  |  |  |  |  |  |  |  |  | 1 | 1-2 | 2-3 | 3-4 | 4-5 | 5-6 | 6-7 | 7-8 |
| 2010 |  |  |  |  |  |  |  |  |  |  |  |  |  |  | 1 | 1-2 | 2-3 | 3-4 | 4-5 | 5-6 | 6-7 |
| 2011 |  |  |  |  |  |  |  |  |  |  |  |  |  |  |  | 1 | 1-2 | 2-3 | 3-4 | 4-5 | 5-6 |
| 2012 |  |  |  |  |  |  |  |  |  |  |  |  |  |  |  |  | 1 | 1-2 | 2-3 | 3-4 | 4-5 |
| 2013 |  |  |  |  |  |  |  |  |  |  |  |  |  |  |  |  |  | 1 | 1-2 | 2-3 | 3-4 |
| 2014 |  |  |  |  |  |  |  |  |  |  |  |  |  |  |  |  |  |  | 1 | 1-2 | 2-3 |
| 2015 |  |  |  |  |  |  |  |  |  |  |  |  |  |  |  |  |  |  |  | 1 | 1-2 |
| 2016 |  |  |  |  |  |  |  |  |  |  |  |  |  |  |  |  |  |  |  |  | 1 |
